# Clinical Outcomes of Partial Injection Underwater Endoscopic Mucosal Resection for Superficial Non‐ampullary Duodenal Epithelial Tumors (With Video)

**DOI:** 10.1002/deo2.70453

**Published:** 2026-10-21

**Authors:** Noboru Misawa, Takuma Higurashi, Yuki Asano, Kojiro Inoue, Hitomi Suzuki, Shigeki Tamura, Takashi Kobayashi, Michihiro Iwaki, Asako Nogami, Sho Hasegawa, Masato Yoneda

**Affiliations:** ^1^ Department of Gastroenterology and Hepatology Yokohama City University School of Medicine Kanagawa Japan

**Keywords:** duodenal neoplasms, endoscopic mucosal resection, endoscopy, submucosal injection, underwater technique

## Abstract

**Objectives:**

Although endoscopic resection is widely performed for superficial non‐ampullary duodenal epithelial tumors (SNADETs), conventional endoscopic mucosal resection (CEMR) may be associated with suboptimal complete resection rates. Partial injection underwater EMR (PI‐UEMR) has been proposed to improve the visualization of distal lesion margins and facilitate en bloc resection. We aimed to evaluate the outcomes of PI‐UEMR for SNADETs, using CEMR as a historical institutional comparator.

**Methods:**

This single‐center observational cohort study included patients who underwent endoscopic mucosal resection for SNADETs between September 2015 and December 2025. Lesions treated with CEMR or PI‐UEMR were analyzed. The primary outcomes were en bloc and R0 resection rates. The secondary outcomes included histological diagnosis and procedure‐related adverse events, including intraoperative perforation, delayed perforation, and delayed bleeding.

**Results:**

In total, 77 lesions in 73 patients were analyzed, including 39 treated with CEMR and 38 treated with PI‐UEMR. The en bloc resection rates were 90.0% and 97.4% in the CEMR and PI‐UEMR groups, respectively. The R0 resection rate was significantly higher in the PI‐UEMR group than in the CEMR group (89.4% vs. 69.2%). Histological diagnoses were similar between the groups. No intraoperative perforation occurred in either group. No procedure‐related adverse events, including delayed perforation or bleeding, were observed in the PI‐UEMR group.

**Conclusions:**

While maintaining a favorable safety profile, PI‐UEMR was associated with a higher R0 resection rate than CEMR in this study. It may represent an effective endoscopic treatment option for SNADETs, particularly when precise visualization of the distal lesion margins is required.

**Trial Registration**: University Hospital Medical Information Network: UMIN000060637

## 1 Introduction

Superficial non‐ampullary duodenal epithelial tumors (SNADETs) are increasingly detected owing to advances in endoscopic imaging and the widespread use of upper gastrointestinal endoscopy [1, 2]. Endoscopic resection is an important treatment option for SNADETs with malignant potential [3, 4]. However, duodenal endoscopic resection remains technically challenging because of the thin duodenal wall, limited maneuverability, and exposure to bile and pancreatic juice, which increase the risk of procedure‐related adverse events [5, 6].

Conventional endoscopic mucosal resection (CEMR) is widely used for SNADETs, particularly for lesions smaller than 20 mm [7, 8]. However, achieving reliable en bloc and R0 resection remains challenging [9]. Piecemeal resection may compromise pathological assessment and is associated with local recurrence [10, 11, 12].

Underwater EMR (UEMR) has been introduced as an alternative approach to overcome some of the limitations of CEMR. By eliminating submucosal injection and using water immersion to float the mucosa, UEMR facilitates snare placement and may reduce technical difficulties in selected cases [13]. Several studies have reported that UEMR is a feasible technique for treating duodenal tumors [14, 15, 16]. Nevertheless, UEMR has inherent limitations. In particular, visualization and control of the distal margin of the lesion may be insufficient under water immersion, which can result in suboptimal en bloc and R0 resection, especially for intermediate‐sized lesions or those in technically challenging locations [17, 18].

To address these unresolved issues, partial injection UEMR (PI‐UEMR) was developed by Takatori et al. as a modification of UEMR aimed at improving resection quality while maintaining procedural safety [19, 20]. In PI‐UEMR, localized submucosal injection is performed selectively on the distal side of the lesion under water to enhance visualization of the distal lesion margin and facilitate stable snare placement, potentially improving en bloc and R0 resection rates.

Although standard UEMR has become an established treatment option for SNADETs, evidence regarding the clinical outcomes of PI‐UEMR remains limited. We therefore aimed to evaluate the clinical outcomes and safety of PI‐UEMR for SNADETs, using CEMR as a historical institutional comparator.

## 2 Methods

### 2.1 Study Design and Setting

This was a single‐center retrospective analysis of an observational cohort of consecutive patients who underwent endoscopic resection for SNADETs between September 2015 and December 2025 at Yokohama City University Hospital (Japan). This study was approved by the Ethics Committee of Yokohama City University Hospital in December 2022 (approval number: F221200004). Eligible patients treated before ethics approval were identified from existing medical records, whereas patients treated after ethics approval were included through continued observational data collection. No study‐specific intervention was performed, and all treatment decisions were made independently of the study protocol. The study protocol complied with the Declaration of Helsinki and the Ethics Guidelines for Clinical Research published by the Ministry of Health, Labor and Welfare, Japan. The Ethics Committee approved the study and granted a waiver of written informed consent, and an opt‐out consent process was employed. The trial was registered with the University Hospital Medical Information Network Clinical Trials Registry (UMIN000060637).

### 2.2 Patients and Lesions

This study included patients with endoscopically diagnosed SNADETs who underwent endoscopic resection during the study period. Clinical data, including endoscopic reports and recorded endoscopic images or videos, were collected from electronic medical records. Patients were eligible for inclusion if they underwent CEMR or PI‐UEMR as the initial treatment for SNADETs. Lesions involving the ampulla of Vater, those with suspected deep submucosal invasion, and those treated with endoscopic submucosal dissection or surgical resection were excluded from the analysis.

Generally, EMR was considered for lesions smaller than 20 mm without endoscopic features suggestive of deep submucosal invasion and that were judged amenable to snare resection. No predefined lesion‐specific criteria were used to select between CEMR and PI‐UEMR. The treatment strategy changed over time at our institution: CEMR was the principal EMR technique from September 2015 through April 2023, whereas PI‐UEMR was introduced in May 2023 and subsequently used through December 2025. Thus, the two techniques were applied during distinct chronological periods rather than being selected concurrently on a case‐by‐case basis. Standard UEMR was performed in only a limited number of patients during the study period and was therefore not included as a comparator because the number of eligible cases was insufficient for a statistically meaningful analysis. Accordingly, CEMR was used as a historical institutional comparator for the exploratory evaluation of PI‐UEMR.

Baseline patient characteristics, including age, sex, and use of antithrombotic agents, were obtained from medical records. Lesion characteristics, including the size, longitudinal location within the duodenum, circumferential location, relationship to the papilla of Vater, and macroscopic type, were evaluated on the basis of endoscopic findings and recorded endoscopic images or videos. The circumferential location was classified as anterior, posterior, medial, or lateral wall. The tumor size was estimated endoscopically using the diameter of the open biopsy forceps or a snare as a reference.

### 2.3 General Information on the Endoscopic Procedure

All the endoscopic procedures were performed using a standard upper gastrointestinal endoscope equipped with a water‐jet function and carbon dioxide insufflation. The endoscopes used included GIF‐Q260J, GIF‐H290T, and GIF‐XZ1200 (Olympus Medical Systems, Tokyo, Japan), which were selected according to the lesion location and endoscopist preference. Transparent distal attachments were used in all patients.

Procedures were performed under sedation with propofol and/or dexmedetomidine with continuous cardiopulmonary monitoring.

Lesion morphology and margins were carefully assessed using white‐light imaging and image‐enhanced endoscopy when necessary. In all the patients, the post‐resection mucosal defect was prophylactically closed using endoscopic clips. All CEMR and PI‐UEMR procedures were performed by two experienced endoscopists (Noboru Misawa and Takuma Higurashi), each of whom had performed >500 therapeutic endoscopic procedures.

### 2.4 Endoscopic Devices

Dedicated electrosurgical snares were used for CEMR and PI‐UEMR. The snares included Captivator II (Boston Scientific, Marlborough, MA, USA) and SnareMaster (Olympus Medical Systems), with the size selected according to the lesion diameter and morphology.

Electrosurgical current was delivered using a high‐frequency generator (VIO 300 or VIO 3; ERBE Elektromedizin, Tübingen, Germany) with standard cutting and coagulation settings for gastrointestinal EMR, in accordance with institutional practice. Normal saline containing diluted epinephrine was used for submucosal injection during CEMR, in accordance with our conventional institutional practice. For PI‐UEMR, sodium alginate solution (Liftal K; Kaigen Pharma Corporation, Osaka, Japan) was used because its higher viscosity and longer‐lasting lifting effect were considered suitable for creating and maintaining a localized elevation on the distal side of the lesion during underwater resection. Diluted epinephrine was added to the injection solution in both techniques. Injections were performed using a standard 23–25‐gauge endoscopic injection needle.

### 2.5 Endoscopic Procedures

CEMR was performed using standard techniques. After submucosal injection to achieve adequate lifting of the lesion, the lesion was resected using a snare with electrocautery. Piecemeal resection was permitted when en bloc resection was considered technically difficult. After resection, the mucosal defect was prophylactically closed using endoscopic clips in all the patients.

PI‐UEMR was performed using the same technique as originally described by Takatori et al. [19, 20], without procedural modification. Briefly, after the duodenal lumen was filled with saline, a localized submucosal injection of sodium alginate solution containing diluted epinephrine and a small amount of indigo carmine was administered selectively to the anal side of the lesion underwater to improve visualization and delineation of the lesion margin. Following partial injection, the lesion was resected underwater using a snare without further submucosal injection. The injection was intentionally limited to the anal side of the lesion and was not performed circumferentially or beneath the entire lesion. PI‐UEMR was distinguished from CEMR based on this procedural protocol and subsequent underwater snare resection, irrespective of the actual extent of submucosal spread of the injected solution. After resection, the mucosal defect was prophylactically closed using endoscopic clips in all the patients (Figure 1a–f). The PI‐UEMR procedure is demonstrated in Video S1.

**FIGURE 1:**
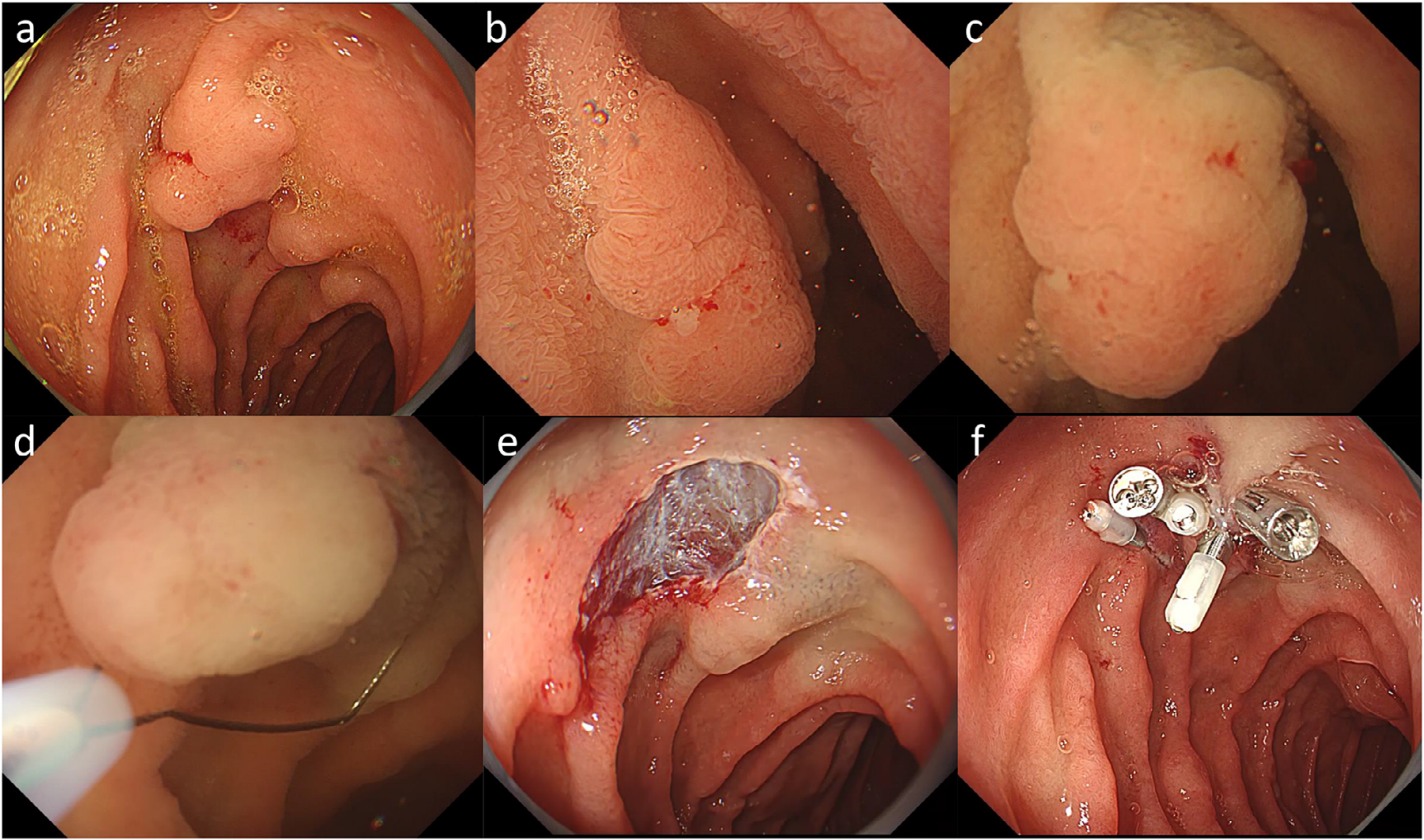
Representative procedure of PI‐UEMR. (a) Endoscopic view of a superficial non‐ampullary duodenal epithelial tumor located in the second portion of the duodenum, proximal to the papilla of Vater, before the procedure. (b) Underwater view after filling the lumen with saline, showing partial collapse of the duodenal lumen and suboptimal visualization of the lesion margin. (c) Partial submucosal injection of sodium alginate solution on the anal side of the lesion to improve visualization of the distal margin. (d) Snaring of the lesion underwater following partial submucosal injection. (e) Mucosal defect after en bloc resection. (f) Complete closure of the mucosal defect using endoscopic clips. PI‐UEMR, partial injection underwater endoscopic mucosal resection.

### 2.6 Outcome Measures

The primary outcomes were en bloc and R0 resection rates. En bloc resection was defined as the removal of the lesion in a single piece, and R0 resection was defined as en bloc resection with histologically negative horizontal and vertical margins.

The secondary outcomes included procedure‐related adverse events, specifically delayed bleeding, intraoperative perforation, and delayed perforation. Delayed bleeding was defined as overt bleeding, melena, or a decrease in the hemoglobin level requiring endoscopic intervention within 14 days after the procedure. Intraoperative perforation was defined as the identification of a full‐thickness defect in the duodenal wall or extraluminal exposure during the endoscopic procedure, requiring endoscopic closure or immediate recognition by the endoscopist. Delayed perforation was defined as clinical or radiological evidence of perforation occurring after completion of the endoscopic procedure, typically presenting with abdominal pain, free air on imaging, or signs of peritonitis confirmed by imaging studies or endoscopic findings within 14 days post‐procedure.

### 2.7 Statistical Analysis

Continuous variables are expressed as mean ± standard deviation or median (interquartile range [IQR]), as appropriate according to their distribution, and categorical variables are expressed as count and percentage. Differences in continuous variables between the CEMR and PI‐UEMR groups were compared using the Student t‐test for normally distributed data or the Mann–Whitney U test for non‐normally distributed data. Categorical variables were compared using the chi‐square test or Fisher exact test, as appropriate. An exploratory subgroup analysis was performed according to lesion size (≤10 and >10–≤15 mm). No lesions measuring >15–<20 mm were included in the present cohort; therefore, this size category could not be analyzed. A two‐sided *p*‐value <0.05 was considered statistically significant, and the statistical analyses were performed using SPSS Statistics (version 24.0; IBM Corp., Armonk, NY, USA).

## 3 Results

### 3.1 Patient and Lesion Characteristics

In total, 39 lesions in 35 patients who were treated with CEMR and 38 lesions in 38 patients who were treated with PI‐UEMR were analyzed (Figure 2). No significant differences were observed between the CEMR and PI‐UEMR groups with respect to age, sex distribution, body mass index, or use of antithrombotic agents (Table 1).

**FIGURE 2:**
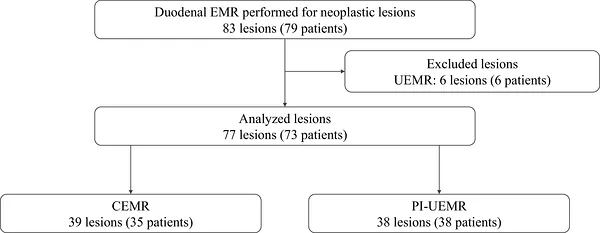
Flow diagram of patient selection and treatment allocation. A total of 83 lesions in 79 patients underwent endoscopic resection for SNADETs during the study period. After exclusion of six lesions treated with UEMR using cold snare polypectomy, 77 lesions in 73 patients were included in the analysis, comprising 39 lesions treated with CEMR and 38 lesions treated with PI‐UEMR. SNADETs, superficial non‐ampullary duodenal epithelial tumors; PI‐UEMR, partial injection underwater endoscopic mucosal resection; CEMR, conventional endoscopic mucosal resection; EMR, endoscopic mucosal resection.

**TABLE 1:** Patient characteristics of the two study groups.

Characteristic	CEMR (*n* = 35)	PI‐UEMR (*n* = 38)	*p*
Age, years, mean (SD)	66.5 (12.3)	66.7 (10.3)	0.928
Sex (M:F)	19:16	28:10	0.095
BMI, kg/m^2^, mean (SD)	22.4 (2.6)	23.7 (3.9)	0.094
Antithrombotic therapy, *n* (%)	4 (11.4)	4 (10.5)	1

BMI, body mass index; CEMR, conventional endoscopic mucosal resection; F, female; M, male; PI‐UEMR, partial injection underwater endoscopic mucosal resection; SD, standard deviation.

The median tumor size was not significantly different between the groups. The distribution of macroscopic type (elevated versus [vs.] depressed) was also comparable between the two groups. Most lesions were located in the second portion of the duodenum in both groups, with no significant differences in distribution across the first, second, and third portions. The distribution of the circumferential lesion location was also comparable between the CEMR and PI‐UEMR groups (*p* = 0.984). Additionally, the relationship between the lesion and the papilla of Vater (upper vs. lower side) did not differ significantly between the groups (Table 2).

**TABLE 2:** Lesion characteristics of the two study groups.

Lesion characteristic	CEMR (*n* = 39)	PI‐UEMR (*n* = 38)	*p*
Size, mm, median (IQR)	10 (5–10)	7 (6–9)	0.260
Macroscopic type (elevated:depressed)	25:14	20:18	0.359
Location, *n* (%)			0.120
First portion	7 (17.9)	2 (5.3)	
Second portion	30 (76.9)	35 (92.1)	
Third portion	2 (5.1)	1 (2.6)	
Circumferential location, *n* (%)			0.984
Anterior wall	7 (17.9)	6 (15.8)	
Posterior wall	10 (25.6)	10 (26.3)	
Medial wall	9 (23.1)	8 (21.1)	
Lateral wall	13 (33.3)	14 (36.8)	
Relationship with papilla of Vater, *n* (%)			0.497
Upper	22 (56.4)	18 (47.4)	
Lower	17 (43.6)	20 (52.6)	

CEMR, conventional endoscopic mucosal resection; IQR, interquartile range; PI‐UEMR, partial injection underwater endoscopic mucosal resection.

### 3.2 Treatment Outcomes and Complications

The en bloc resection rate was high in both groups. The R0 resection rate was significantly higher in the PI‐UEMR group than in the CEMR group. Results of the exploratory subgroup analysis stratified by the lesion size are shown in Table S1. No lesions measuring >15–<20 mm were included in the cohort. Given the limited number of lesions in the >10–≤15–mm subgroup, these findings should be interpreted as exploratory.

Histopathological diagnoses did not differ significantly between the groups, with the majority of lesions in both groups being diagnosed as adenomas, whereas adenocarcinomas were identified in the remaining lesions.

Regarding procedure‐related adverse events, no intraoperative perforations occurred in either group. The treatment outcomes and complications are summarized in Table 3. No local recurrence was observed in either group during the follow‐up period.

**TABLE 3:** Treatment outcomes and complications of the two study groups.

Outcome/Complication	CEMR (*n* = 39)	PI‐UEMR (*n* = 38)	*p*
En bloc resection, *n* (%)	35 (90.0)	37 (97.4)	0.358
R0 resection, *n* (%)	27 (69.2)	34 (89.4)	0.047
Histological diagnosis, *n* (%)			0.807
Adenoma	28 (71.8)	26 (68.4)	
Adenocarcinoma	11 (28.2)	12 (31.6)	
Intraoperative perforation, *n* (%)	0 (0)	0 (0)	1
Delayed perforation, *n* (%)	1 (2.6)	0 (0)	1
Delayed bleeding, *n* (%)	2 (5.1)	0 (0)	0.494
Follow‐up period, months, median (IQR)	38.5 (3–71)	13 (4–25)	0.058
Local recurrence, *n* (%)	0 (0)	0 (0)	1

CEMR, conventional endoscopic mucosal resection; IQR, interquartile range; PI‐UEMR, partial injection underwater endoscopic mucosal resection.

## 4 Discussion

Here, PI‐UEMR was associated with a higher R0 resection rate than CEMR, whereas the en bloc resection rate was numerically higher but did not differ significantly between the groups. A favorable safety profile was observed in the PI‐UEMR group. These findings indicate favorable histologically complete resection and safety outcomes with PI‐UEMR in this historical comparison.

Previous studies have reported en bloc resection rates of approximately 80%–90% and R0 resection rates of approximately 60%–80% with CEMR for SNADETs [7, 8, 18]. In this study, the R0 resection rate in the CEMR group was 69.2%, which is consistent with findings of previous studies. By contrast, a higher R0 resection rate was observed in the PI‐UEMR group. However, given the historical nature of the comparison, this difference cannot be attributed solely to the procedural technique.

Recently, UEMR has emerged as an alternative technique for the treatment of SNADETs and has been reported to achieve favorable clinical outcomes with low complication rates. Several studies have demonstrated en bloc resection rates of approximately 85%–95% with UEMR, with acceptable safety profiles [17, 18]. However, the reported R0 resection rate for UEMR varies across studies and ranges from approximately 50%–60%, indicating that histologically complete resection is not always achieved [17, 18]. To address this limitation, Takatori et al. proposed PI‐UEMR, which combines UEMR with localized submucosal injection on the anal side of the lesion [19, 20]. In their feasibility study and subsequent propensity score–matched analysis, PI‐UEMR demonstrated improved R0 resection rates compared with UEMR.

Although standard UEMR would be a clinically relevant comparator for PI‐UEMR, only a limited number of patients underwent standard UEMR at our institution during the study period, precluding a statistically meaningful direct comparison. Before the introduction of PI‐UEMR in May 2023, CEMR had been the principal EMR technique for SNADETs and was therefore used as a historical institutional comparator in the present exploratory analysis. Thus, the higher R0 resection rate observed with PI‐UEMR compared with CEMR should not be interpreted as evidence of superiority over standard UEMR. The findings of the present study are consistent with those of previous studies showing favorable resection outcomes with PI‐UEMR. However, direct comparative studies between PI‐UEMR and standard UEMR are required to determine whether PI‐UEMR provides additional clinical benefit over UEMR.

In PI‐UEMR, a limited submucosal injection on the anal side of the lesion creates a localized elevation that can serve as an anchor point for the tip of the snare. This structural effect may facilitate stable snaring that reliably includes the distal margin, thereby improving the likelihood of achieving R0 resection. Additionally, by restricting the injection to a small area, PI‐UEMR avoids excessive mucosal elevation, which may interfere with snare capture during CEMR. These technical characteristics may explain the favorable resection outcomes observed here and in previous studies evaluating PI‐UEMR for SNADETs.

However, this study has some limitations. This was a nonrandomized, single‐center observational study, and residual selection bias and unmeasured confounding cannot be excluded. Moreover, the two groups cannot be considered directly comparable in the same manner as contemporaneous treatment groups. The sample size was small and may not have been sufficient to detect rare adverse events or to generalize the findings to all clinical settings. The treatment groups were also derived from different chronological periods; thus, various factors and historical bias may have influenced the observed differences in the treatment outcomes. Although standard UEMR was performed at our institution during the study period, the number of eligible cases was limited and insufficient for a statistically meaningful direct comparison with PI‐UEMR. Hence, the present findings should be interpreted as an exploratory comparison with our historical CEMR cohort and do not establish the superiority of PI‐UEMR over standard UEMR. Larger prospective studies directly comparing PI‐UEMR with standard UEMR are warranted. Different submucosal injection solutions were also used for CEMR and PI‐UEMR. Therefore, the observed differences in resection outcomes may have been influenced not only by the procedural technique but also by differences in the injection material, and these effects could not be separated in the present study. Furthermore, in small lesions, particularly those measuring <10 mm, the injected solution may spread beyond the intended distal side and extend beneath a substantial portion or the entirety of the lesion. Thus, the technical distinction between PI‐UEMR and CEMR may be less apparent in such lesions, although the procedures were classified according to the intended injection strategy and whether snare resection was performed underwater. Finally, the median follow‐up period was shorter in the PI‐UEMR group than in the CEMR group, although the difference did not reach statistical significance. This difference reflects, at least in part, the later introduction of PI‐UEMR. Accordingly, the absence of local recurrence, particularly in the PI‐UEMR group, should be interpreted cautiously. Longer‐term follow‐up is needed to more reliably assess local recurrence after PI‐UEMR.

In conclusion, PI‐UEMR showed favorable histologically complete resection and safety outcomes in this historical comparison with CEMR. Therefore, PI‐UEMR may offer an effective endoscopic option for lesions in which accurate visualization of the distal margin is technically challenging.

## Author Contributions

**Noboru Misawa**, **Shigeki Tamura**, and **Takuma Higurashi** conceived the study. **Noboru Misawa** and **Takuma Higurashi** performed CEMR and PI‐UEMR. **Kojiro Inoue**, **Hitomi Suzuki**, **Takashi Kobayashi**, **Michihiro Iwaki**, **Asako Nogami**, and **Sho Hasegawa** recruited the study participants. **Noboru Misawa**, **Takuma Higurashi**, and **Masato Yoneda** conducted the data analysis and interpretation. All authors have read the final manuscript and have approved its submission for publication.

## Funding

The authors have nothing to report.

## Ethics Statement

**Approval of the research protocol by an Institutional Reviewer Board**: The study was approved by the Ethics Committee of Yokohama City University Hospital in December 2022.

## Consent

The requirement for written informed consent was waived, and an opt‐out consent process was employed. Information about the study was disclosed on the institution's website, and participants were given the opportunity to decline participation.

## Conflicts of Interest

The authors declare no conflicts of interest.

## Supporting information




**Video S1**: Representative PI‐UEMR procedure for a SNADET, demonstrating targeted submucosal injection on the anal side of the lesion followed by snare resection underwater without additional submucosal injection. SNADET, superficial non‐ampullary duodenal epithelial tumor; PI‐UEMR, partial injection underwater endoscopic mucosal resection.

**Supporting File**: Deo270453‐sup‐0002‐tableS1.docx.
