# Supplementary material for: Clinical Outcomes of Partial Injection Underwater Endoscopic Mucosal Resection for Superficial Non‐ampullary Duodenal Epithelial Tumors (With Video)
Source: DEN Open. 2026 Oct 21;7(1):e70453. doi: 10.1002/deo2.70453 (PMC13685527; doi:10.1002/deo2.70453)
Supplement: Supplementary file 1 [file DEO2-7-e70453-s002.docx]

**Table S1** Results of the exploratory subgroup analysis according to the lesion size

| Lesion size | Outcome | CEMR | PI-UEMR | P |
| --- | --- | --- | --- | --- |
| ≤10 mm | En bloc resection, n/N (%) | 29/30 (96.7) | 31/31 (100) | 0.492 |
|  | R0 resection, n/N (%) | 22/30 (73.3) | 29/31 (93.5) | 0.043 |
| >10 to ≤15 mm | En bloc resection, n/N (%) | 6/9 (66.7) | 6/7 (85.7) | 0.585 |
|  | R0 resection, n/N (%) | 5/9 (55.6) | 5/7 (71.4) | 0.633 |

PI-UEMR, partial injection underwater endoscopic mucosal resection; CEMR, conventional endoscopic mucosal resection
